# Facts about Gold

**Published:** 1881-12

**Authors:** 


### FACTS ABOUT GOLD.

From a mass of literature concerning the future of gold, and in which the deductions made by the writers are as far as the poles asunder, the following facts are gathered, namely: The annual supply of the metal is $118,000,000; the price, $18.96 per ounce, has not varied for 160 years; since the discovery of America by Columbus, the mines of Mexico, California, British Columbia and other parts of North America have added $4,369,000,000 to the stock of gold in the world; $8,000,000,000 is the estimated value of the metal now in use; the gold coinage has increased from $77,000,000 in 1848 to $182,000,000 in 1880. It is also stated that the annual product in 1492 was $100,000; that it gradually increased to $17,000,000 in 1800; that it reached $236,000,000 in 1853; and that $8,000,000 are annually consumed in the arts and lost by fire and shipwreck. The total value of the auriferous product from the earliest times is said to be $14,000,000,000, of which $8,106,000,000 have been added within 39 years, and of this latter amount $7,895,000,000 are still in existence, one-fifth being furnished by North American mines. The great increase in the supply of the metal since the discoveries in California may have had some effect on the prices of merchandise. But it appears, from a comparison of prices in 1850 and 1880, that the advance has not been uniform, and a close inspection of the conditions reveals that there have been causes other than the abundant supply of money which have inflated values, which, by the way, have fluctuated widely during the last 30 years.

